# Liver resection for a congenital intrahepatic portosystemic shunt in a child with hyperammonemia and hypermanganesemia: a case report

**DOI:** 10.1186/s40792-020-00838-5

**Published:** 2020-04-17

**Authors:** Yuichi Takama, Tetsuro Nakamura, Kenji Santo, Akihiro Yoneda

**Affiliations:** 1 Department of Pediatric Surgery, Osaka City General Hospital, Osaka, Japan; 2 Department of Pediatric Surgery, Kindai University Nara Hospital, Nara, Japan

**Keywords:** Intrahepatic portosystemic shunt, Liver resection, Pediatric, Hyperammonemia, Hypermanganesemia, Congenital portosystemic shunt

## Abstract

**Background:**

Congenital portosystemic shunt (CPSS) is a rare malformation that leads to hyperammonemia, hypermanganesemia, and various symptoms. CPSSs are divided into intrahepatic and extrahepatic shunts. In patients with persistent CPSS including an intrahepatic portosystemic shunt (IPSS), early intervention to occlude the shunt reverses the associated complications.

**Case presentation:**

The patient was a 1-year-and-7-month-old girl. She presented with hypergalactosemia and elevation of blood ammonia level (75 μg/dL) and total bile acid levels (68.2 μmol/L) during the neonatal period. Two IPSSs were detected using ultrasound and enhanced computerized tomography. Magnetic resonance imaging (MRI) at 1 year and 3 months of age showed abnormally high signal intensity in the pallidum of her brain. Spontaneous closure was not observed. We performed a right hepatectomy at 1 year and 7 months of age. The portal vein pressure was 16 mmHg after temporary occlusion of the right portal vein. Blood ammonia and serum manganese levels decreased immediately after the operation. The abnormal signal on brain MRI disappeared. She had a favorable course with no sign of recurrence of IPSS 5 years postoperatively.

**Conclusion:**

Liver resection for an IPSS to control the symptoms of a portosystemic shunt is reasonable in a child for whom interventional radiological treatment is not indicated.

## Background

Congenital portosystemic shunt (CPSS) is a rare malformation that leads to hyperammonemia, hypermanganesemia, hepatic encephalopathy, pulmonary hypertension, hepatopulmonary syndrome, and liver tumor [1, 2]. CPSSs are divided into intrahepatic and extrahepatic shunts [3]. Patients with CPSS present with a wide spectrum of symptoms and complications that may present with unexplained neurocognitive dysfunction and other behavioral issues due to low-grade hepatic encephalopathy that may occur during life [2]. As congenital intrahepatic portosystemic shunts (IPSSs) have a tendency to spontaneously close by 1 year of age with resolution of symptoms, an IPSS that is diagnosed prenatally or during early infancy does not necessarily require definitive treatment [2]. In patients with persistent CPSSs including IPSSs, early intervention to occlude the shunt reverses associated complications. Although Papamichail et al. reported liver resection for the treatment of IPSS in adult patients in 2016 [4], there have been no reports in a pediatric case.

## Case presentation

The patient was a 1-year-and-7-month-old girl. Written informed consent was obtained from the patient’s family. The prenatal and perinatal courses were uneventful. She was born at 40 weeks with normal birth weight. Neonatal mass screening tests on the 4th day of life showed hypergalactosemia (10.78 mg/dL). Although the levels of aspartate aminotransferase (AST) and alanine aminotransferase (ALT) were within normal limits, elevations of blood ammonia level (75 μg/dL) and total bile acid level (68.2 μmol/L) were observed on the 10th day of life. There was no sign of enzyme deficiency to explain galactosemia. Initially, she received no treatment other than careful observation. Subsequent ultrasound at 8 months of age revealed an IPSS with a communication between the right portal vein and the right hepatic vein at a peripheral location of the right liver lobe. She was admitted to our hospital with an IPSS. Her psychomotor development was within normal limits.

**Fig. 1:**
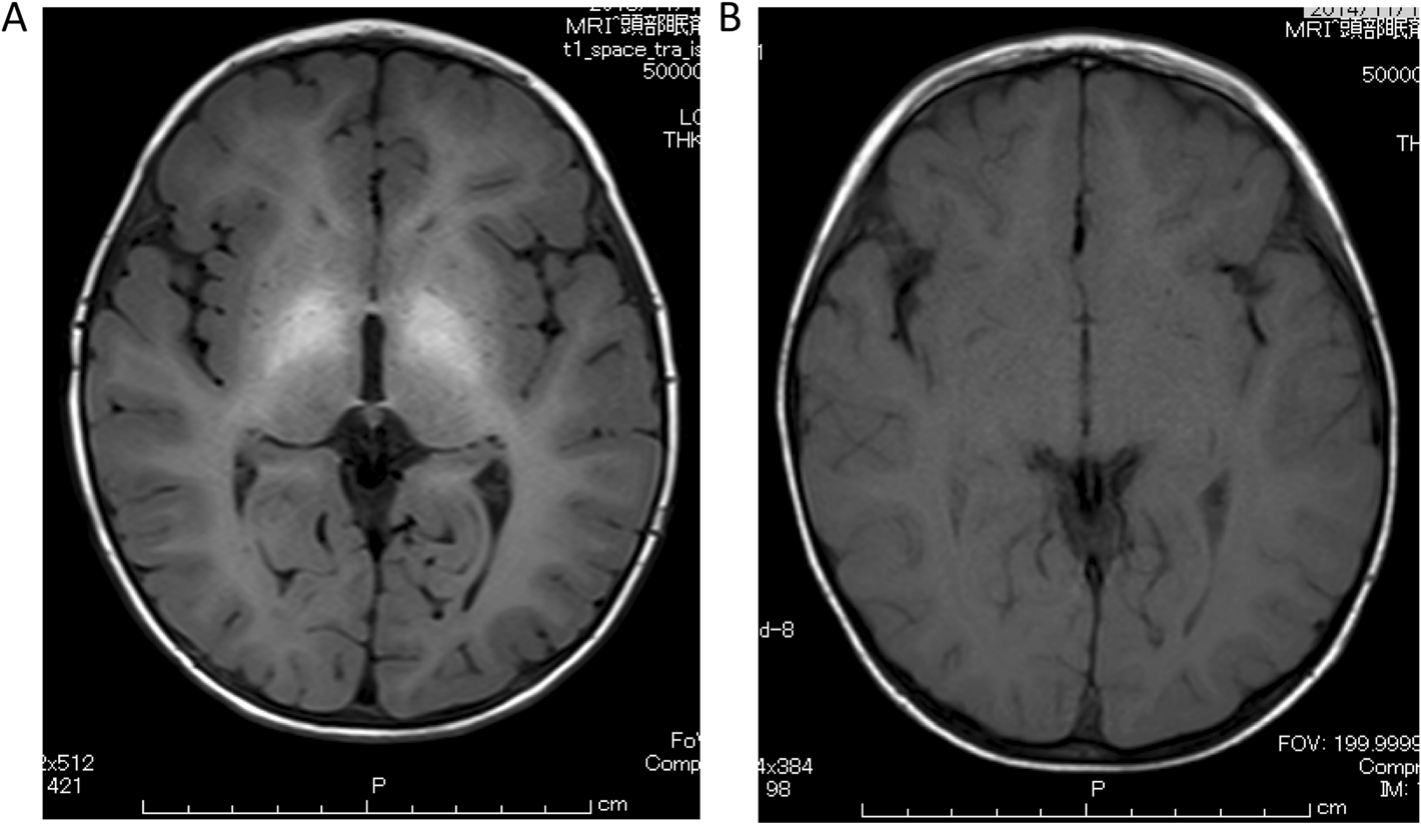
Magnetic resonance imaging of brain. **a** Magnetic resonance imaging (MRI) of the brain at the age of 1 year and 3 months, revealing high intensity of the pallidum. **b** MRI at 8 months postoperatively, revealing normalization of the high intensity of the pallidum

**Fig. 2:**
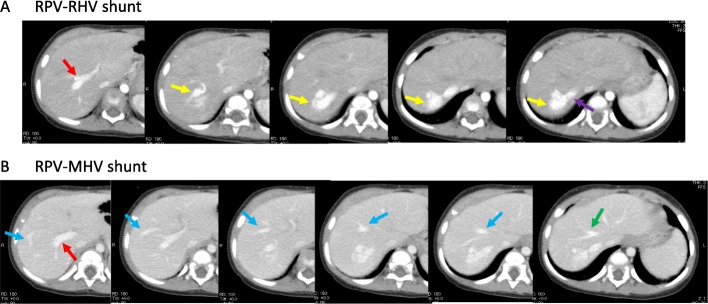
Enhanced computed tomography at 1 year of age. **a** Enhanced computed tomography (CT) revealing a shunt vessel (yellow arrow) between the right portal vein (RPV) (red arrow) and right hepatic vein (RHV) (purple arrow). The shunt vessel (yellow arrow) presented a nidus-like form. **b** Enhanced CT revealing a shunt vessel (blue arrow) between the right portal vein (RPV) (red arrow) and the middle hepatic vein (MHV) (green arrow)

At 1 year of age, there was no sign of spontaneous closure of IPSS. Laboratory tests showed elevated levels of total bile acid (116 μmol/L), hyperammonemia (105 μg/dL), and hypermanganesemia (3.7 μg/dL). Magnetic resonance imaging (MRI) with T1-weighted images at the age of 1 year and 3 months showed abnormal high signal intensity in the pallidum of the brain (Fig. 1a). Detailed examination of the IPSS using enhanced computerized tomography (CT) at 1 year of age revealed two IPSSs (Fig. 2). One was a nidus forming a shunt between the right portal vein and the right hepatic vein; the other was between the right posterior portal vein and the middle hepatic vein at the peripheral location of the right liver lobe. Subsequently, there was no improvement of hyperammonemia and hypermanganesemia. Spontaneous closure was not observed. The possibility of closure of the IPSSs by interventional radiologic techniques was ruled out because of the nidus forming the shunt and the multiple shunts. Therefore, we performed a right hepatectomy at 1 year and 7 months of age.

**Fig. 3:**
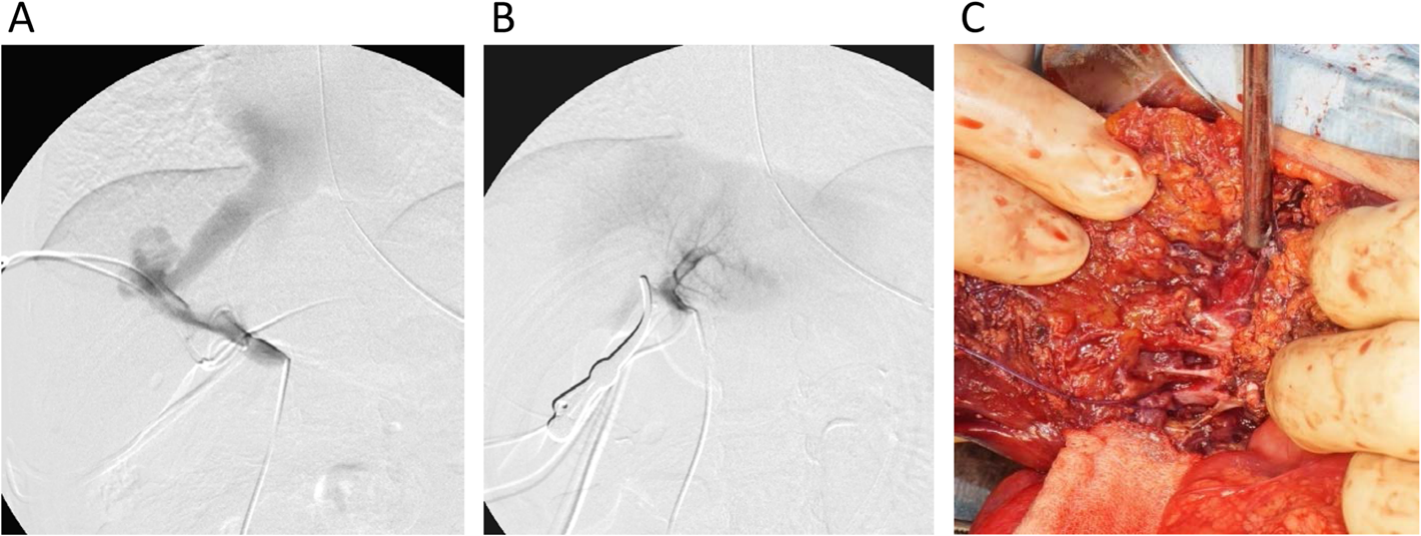
Intraoperative findings. **a** Portography and measurement of portal vein pressure were performed. Before temporary occlusion of the right portal vein, portal vein pressure was 13 mmHg and portography showed almost all portal vein flow draining into the hepatic vein through the IPSS. **b** After the temporary occlusion of the right portal vein, the portal vein pressure was 16 mmHg, and portography showed good contrast of left portal vein, intrahepatic portal vein, and hepatic vein and no IPSS in the left lobe. **c** A collection of multiple vessels was observed that were thought to be shunt vessels during resection of liver parenchyma

During laparotomy, a catheter was inserted through the peripheral portion of the superior mesenteric vein and placed in the portal vein. We dissected the portal vein, and tested temporary occlusion of the right portal vein using vascular forceps. The portal vein pressure was measured, and portography was performed using this catheter before and after the temporary occlusion. The portal vein pressure was 13 mmHg before temporary occlusion of the right portal vein. Portography showed almost all portal vein flow draining into the hepatic vein through the IPSS before occlusion of the right portal vein. Although the left portal vein was not detected before occlusion, portography showed good contrast of the left portal vein, intrahepatic portal vein, and hepatic vein and showed no IPSS in the left lobe after temporary occlusion of the right portal vein (Fig. 3a, b). We evaluated the portal vein pressure and congestion of the intestine after temporal occlusion for about 20 min. The portal vein pressure was 16 mmHg after temporary occlusion of the right portal vein. There was no sign of intestinal wall edema and redness, as would accompany intestinal congestion.

**Fig. 4:**
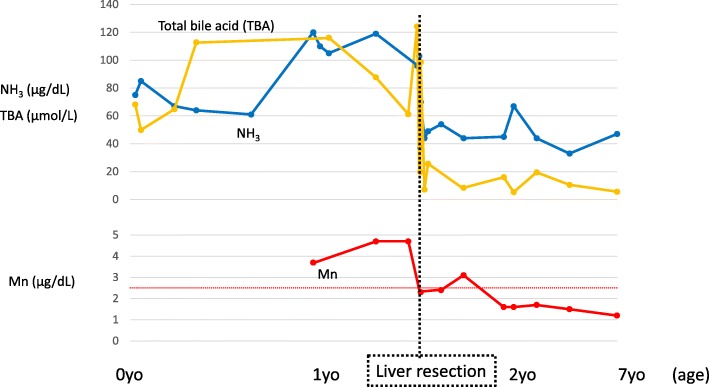
Changes in laboratory data. Upper panel showing changes in ammonia and total bile acid levels. Lower panel showing changes in manganese levels

Based on these intraoperative findings, we subsequently performed right hepatectomy. Liver parenchyma was resected along the right side of the middle hepatic vein. A collection of multiple vessels was observed that were thought to be shunt vessels during resection of liver parenchyma (Fig. 3c). No histopathological findings suggesting hemangiomas were found. Dilated blood vessels were observed, but the histopathological findings could not accurately reveal the dilated vessels as IPSSs. The intraoperative and postoperative courses were uneventful. There was no sign of postoperative liver failure, and ammonia and manganese levels decreased immediately after the operation (Fig. 4). An MRI of the brain 8 months after the operation (at 2 years and 5 months old) revealed that the abnormal high signal intensity in the pallidum had disappeared (Fig. 1b). The patient had no sign of recurrence of IPSS and no elevation of blood ammonia level and serum manganese level 5 years postoperatively (Fig. 4).

## Discussion

We performed liver resection for a congenital IPSS in a child with hyperammonemia and hypermanganesemia. CPSS is a rare malformation that leads to a wide spectrum of symptoms and complications, including hyperammonemia, hypermanganesemia, hepatic encephalopathy, pulmonary hypertension, hepatopulmonary syndrome, and liver tumor [1, 2]. IPSS has been further classified into four types by Park et al., based on the morphology of the shunts [5], namely those that include a single vessel communication: between a main branch of the portal vein and inferior vena cava (type 1); in a peripheral location in one segment (type 2); through an aneurysm (type 3); or have multiple small communications distributed diffusely in both lobes (type 4) [5, 6]. Our patient had a combination of types 2 and 3.

Hepatic encephalopathy, hepatopulmonary syndrome, and pulmonary hypertension are the most prominent manifestations caused by long-term portosystemic shunting and are more commonly observed in children [7]. Children with CPSS may present with unexplained neurocognitive dysfunction and other behavioral issues due to low-grade hepatic encephalopathy; this accounts for between 17% and 30% of cases [2, 8]. Other manifestations include learning disabilities, extreme fatigability, seizures, and failure to thrive, associated with elevated arterial ammonia levels in the majority of cases [2]. The likelihood of encephalopathy increases with age and is related to shunt flow [2, 8].

As congenital IPSSs have a tendency to spontaneously close by 1 year of age with resolution of symptoms, an IPSS that is diagnosed prenatally or during early infancy does not necessarily require definitive treatment [2]. Until 1 year of age, a wait-and-see policy is the initial recommended treatment of IPSS. Spontaneous closure of an IPSS is more often seen in girls, in the presence of multiple shunts, and in children with neonatal cholestasis [9]. Our patient showed no tendency toward spontaneous closure. For children with a persistent IPSS, the optimal timing of treatment has not been defined [2]. Some review papers suggest that, even in the absence of overt symptoms, early intervention may prevent hepatopulmonary syndrome and other pulmonary complications as well as neurodevelopmental delay, and may allow the progress of intellectual and psychosocial development [1, 2, 4, 7, 10]. Papamichail et al. proposed that all shunts that persist after the first year of life should be closed without waiting for complications to develop [2].

The basic principle of intervention is to disrupt the abnormal communication between portal and systematic circulation and to restore portal flow to the liver [2]. Interventional radiology is a minimally invasive procedure thought to be the first-line treatment for shunt occlusion. A vascular plug (e.g., Amplatzer) and embolization were used as occlusive materials [2, 11]. Catheter insertion for the treatment of IPSS was performed via a transhepatic route, a transcaval route using internal jugular vein access, and a transileocolic route with minilaparotomy [2, 12]. Gupta et al. reported the embolization of IPSSs at age 14 months [13]. If possible, interventional radiology should be the first-line treatment for shunt occlusion. The choice of a radiological or surgical approach depends on local expertise and shunt anatomy and size [2].

Although surgical ligation of the portal vein or hepatectomy has been replaced by less invasive interventional radiology [14], there are few reports of liver resection for the treatment of IPSS. Papamichail et al. proposed an intervention protocol of CPSS in which liver resection would be performed to treat large intrahepatic multifocal shunts not amenable to interventional radiology in cases of previous failed radiological intervention or where the malignant tumor has developed [2, 4]. Kitami reported the portal vein ligation without the resection of liver parenchyma [15]. Although the portal vein ligation without the resection of liver parenchyma is easier than hepatectomy, we are concerned about the possibility of the liver abscess by an ischemic change and the recurrence of IPSS in unresected liver parenchyma.

In our case, we chose early intervention, because the patient already exhibited a manifestation of hypermanganesemia in her brain at 1 year and 3 months of age. She had two IPSSs in the right liver lobe, there was no appropriate occlusive device of a suitable size to fit her body and shunt vessel at that time. There was limited experience with embolization by interventional radiology for IPSS at infancy or early childhood at our institution. For these reasons, we preferred liver resection with interventional radiology and performed surgery at 1 year and 7 months of age. Although Papamichail et al. reported liver resection for an IPSS in an adult case in 2016 [4], there have been no previous reports in children. The short- and long-term postoperative courses were uneventful.

## Conclusion

Liver resection for an IPSS to control the symptoms of a portosystemic shunt is reasonable in a child for whom interventional radiological treatment is not indicated.

## Data Availability

Not applicable.

## Abbreviations

CPSS: Congenital portosystemic shunt
IPSS: Intrahepatic portosystemic shunt
AST: Aspartate aminotransferase
ALT: Alanine aminotransferase
MRI: Magnetic resonance imaging
CT: Computerized tomography

## References

[1] Takama Y, Ueno T, Umeda S, Saka R, Tazuke Y, Okuyama H (2019). Laparoscopic ligation of a congenital extrahepatic portosystemic shunt for children with hyperammonemia: a single-institution experience. Surg Today.

[2] Papamichail M, Pizanias M, Heaton N (2018). Congenital portosystemic venous shunt. Eur J Pediatr.

[3] Morgan G, Superina R (1994). Congenital absence of the portal vein: two cases and a proposed classification system for portasystemic vascular anomalies. J Pediatr Surg.

[4] Papamichail M, Ali A, Quaglia A, Karani J, Heaton N (2016). Liver resection for the treatment of a congenital intrahepatic portosystemic venous shunt. Hepatobiliary & Pancreatic Diseases International.

[5] Park JH, Cha SH, Han JK, Han MC (1990). Intrahepatic portosystemic venous shunt. AJR Am J Roentgenol.

[6] Senocak E, Oguz B, Edguer T, Cila A (2008). Congenital intrahepatic portosystemic shunt with variant inferior right hepatic vein. Diagn Interv Radiol.

[7] Sokollik C, Bandsma RH, Gana JC, van den Heuvel M, Ling SC (2013). Congenital portosystemic shunt: characterization of a multisystem disease. J Pediatr Gastroenterol Nutr.

[8] Stringer MD (2008). The clinical anatomy of congenital portosystemic venous shunts. Clin Anat.

[9] Paganelli M, Lipsich JE, Sciveres M, Alvarez F (2015). Predisposing factors for spontaneous closure of congenital portosystemic shunts. J Pediatr.

[10] Bernard O, Franchi-Abella S, Branchereau S, Pariente D, Gauthier F, Jacquemin E (2013). Congenital portosystemic shunts in children: recognition, evaluation, and management. Semin Liver Dis.

[11] Power AH, Bjarnason H (2012). Large spontaneous intrahepatic portal-systemic venous shunt treated with coil and Amplatzer vascular plug embolization. Perspect Vasc Surg Endovasc Ther.

[12] Tanoue S, Kiyosue H, Komatsu E, Hori Y, Maeda T, Mori H (2003). Symptomatic intrahepatic portosystemic venous shunt: embolization with an alternative approach. AJR Am J Roentgenol.

[13] Gupta V, Kalra N, Vyas S, Sodhi KS, Thapa BR, Khandelwal N (2009). Embolization of congenital intrahepatic porto-systemic shunt by n-butyl cyanoacrylate. Indian J Pediatr.

[14] Grimaldi C, Monti L, Falappa P, d’Ambrosio G, Manca A, de Ville de Goyet J (2012). Congenital intrahepatic portohepatic shunt managed by interventional radiologic occlusion: a case report and literature review. J Pediatr Surg.

[15] Kitami Y, Usui Y, Rai F, Tominaga S, Hashino H (1985). author_in_Japanese. A surgical case of portal systemic encephalopathy due to an enormous port-hepatic venous shunt. The journal of the Japanese Practical Surgeon Society.

